# Nonpathogenic SIV and Pathogenic HIV Infections Associate with Disparate Innate Cytokine Signatures in Response to *Mycobacterium bovis* BCG

**DOI:** 10.1371/journal.pone.0158149

**Published:** 2016-08-09

**Authors:** Melanie A. Gasper, Shameek P. Biswas, Bridget S. Fisher, Stephanie C. Ehnert, David R. Sherman, Donald L. Sodora

**Affiliations:** 1 University of Washington Pathobiology Graduate Program, Seattle, Washington, United States of America; 2 Center for Infectious Disease Research, formerly Seattle Biomedical Research Institute, Seattle, Washington, United States of America; 3 Yerkes National Primate Research Center, Atlanta, Georgia, United States of America; Emory University School of Medicine, UNITED STATES

## Abstract

Infections with mycobacteria, including *Mycobacterium tuberculosis* (Mtb) and *Mycobacterium bovis (M*. *bovis)* BCG, are a leading cause of morbidity and mortality for HIV-infected persons. In contrast to HIV, nonpathogenic SIV infections of sooty mangabeys are characterized by a lack of clinical disease including an absence of opportunistic infections. The goal of this study was to identify innate immune responses to *M*. *bovis* BCG maintained during nonpathogenic lentiviral infections through a comparison of functional responses during pathogenic HIV or nonpathogenic SIV infections. Monocytes were evaluated for their ability to express key anti-mycobacterial cytokines TNF-α and IL-12 following a six-hour *ex vivo* BCG exposure. While HIV-infection was associated with a decreased percentage of IL-12-producing monocytes, nonpathogenic SIV-infection was associated with an increased percentage of monocytes producing both cytokines. Gene expression analysis of PBMC following *ex vivo* BCG exposure identified differential expression of NK cell-related genes and several cytokines, including IFN-γ and IL-23, between HIV-infected and control subjects. In contrast, SIV-infected and uninfected-control mangabeys exhibited no significant differences in gene expression after BCG exposure. Finally, differential gene expression patterns were identified between species, with mangabeys exhibiting lower IL-6 and higher IL-17 in response to BCG when compared to humans. Overall, this comparison of immune responses to *M*. *bovis* BCG identified unique immune signatures (involving cytokines IL-12, TNF-α, IL-23, IL-17, and IL-6) that are altered during HIV, but maintained or increased during nonpathogenic SIV infections. These unique cytokine and transcriptome signatures provide insight into the differential immune responses to Mycobacteria during pathogenic HIV-infection that may be associated with an increased incidence of mycobacterial co-infections.

## Introduction

*M*. *bovis* Bacillus Calmette-Guérin (BCG), a live-attenuated vaccine against *M*. *tuberculosis* (Mtb), is administered to infants in settings with a high prevalence of tuberculosis. The BCG vaccine is generally safe and efficacious in preventing mortality caused by Mtb in immunocompetent infants and small children [1, 2]. However, it also presents unique challenges in the context of HIV infection as HIV-infected infants have a dramatically increased risk of disseminated BCG disease following vaccination. This finding prompted the WHO to advise against BCG vaccination of infants with a pre-existing HIV infection due to its opportunistic potential in these persons [3, 4].

Nonhuman primate species serve as models of mycobacterial infection, in which a synergistic disease outcome has been shown when BCG is combined with pathogenic simian immunodeficiency virus (SIV) infection in Rhesus macaques [5, 6], successfully modeling the immunopathology observed during HIV/Mtb coinfection of humans. In contrast to the pathogenic outcome observed in Asian macaques, SIV infection of African monkey species is generally associated with a nonpathogenic outcome. The absence of clinical disease in natural host species, such as sooty mangabeys, has been attributed to a co-evolution with their species-specific viruses that has occurred over at least the last 30,000 years [7]. This prolonged virus-host interaction contrasts the relatively recent introduction of HIV and pathogenic SIV into humans and macaques, respectively. One important immunologic difference between non-pathogenic and pathogenic SIV/HIV infections is the presence of the chronic phase systemic immune activation during pathogenic infections that impacts functionality of numerous immune cell subsets, including innate cells [8–10].

Monocytes are critical players in the innate immune response to mycobacteria, and play a role in both the initial bacterial containment as well as the initiation of an adaptive immune response. Although circulating monocytes are relatively refractory to infection with HIV-1 [11], several of their functions important for containing mycobacteria (including phagocytosis, intracellular killing, and cytokine production) become altered during chronic HIV-1 [12–17] and pathogenic SIV infections [18]. One important aspect of the monocyte functional response to mycobacteria is the production of proinflammatory cytokines, including TNF-α and IL-12. The pivotal role for both TNF-α and IL-12 in initiating and maintaining the immune response to mycobacteria has been made evident through gene knockouts in animal models, population-level studies of genetic polymorphisms, and through an increase in mycobacterial infections following targeted immune therapies that block production or function of TNF-α and IL-12 [19–24]. Taken together, these studies implicate altered TNF-α and IL-12 production and/or function in the reduced immune control of mycobacteria. The studies described here demonstrate altered expression of transcripts associated with different immune cell subsets and a number of cytokines between HIV-infected subjects and controls, including TNF-α, IL-12, IL-23 and IFN-γ, as well as between humans and mangabeys (IL-6 and IL-17). Overall, this work provides a novel approach to understanding into innate immune cell alterations important for BCG responses following pathogenic or nonpathogenic lentiviral infection, and yields insight into mechanisms by which HIV-infected individuals become susceptible to opportunistic mycobacterial coinfections.

## Materials and Methods

### Ethics Statement

All protocols involving human subjects were approved by the Western Institutional Review Boards (IRB protocol #20092089) and written informed consent was obtained from all study participants. Blood from chronically infected, antiretroviral therapy (ART)-naive donors was obtained from the University of Washington Center for AIDS Research Madison Clinic. All blood was obtained anonymously and analyzed without knowledge of patient identifying information.

All animal experimentation was conducted following guidelines established by the Animal Welfare Act and the NIH for housing and care of laboratory animals and performed in accordance with Institutional regulations after review and approval by the Institutional Animal Care and Usage Committees at the Yerkes National Primate Research Center (YNPRC). All efforts were made to minimize suffering. All the blood obtained from sooty mangabeys housed at the Yerkes National Primate Research Center, which is accredited by American Association of Accreditation of Laboratory Animal Care. Sooty Mangabeys are fed standard monkey chow (Jumbo Monkey Diet 5037, Purina Mills, St Louis, MO) twice daily. Consumption is monitored and adjustments are made as necessary depending on sex, age, and weight so that animals get enough food with minimum waste. SIV infected and some uninfected sooty mangabeys were housed in adjoining individual primate cages allowing social interactions, under controlled conditions of humidity, temperature and light (12-hour light/12-hour dark cycles). The YNPRC enrichment plan employs several general categories of enrichment. Animals have access to more than one category of enrichment. IACUC proposals include a written scientific justification for any exclusions from some or all parts of the plan. This study was performed in strict accordance with the recommendations in the Guide for the Care and Use of Laboratory Animals of the National Institutes of Health, a national set of guidelines in the U.S. and also to international recommendations detailed in the Weatherall Report (2006). This work received prior approval by the Institutional Animal Care and Use Committee (IACUC) of Yerkes National Primate Research Center's IACUC approved protocol (IACUC #2000280)). Appropriate procedures were performed to ensure that potential distress, pain, discomfort and/or injury was limited to that unavoidable in the conduct of the research plan. The sedative Ketamine (10 mg/kg) and/or Telazol (4 mg/kg) were applied as necessary for blood collections and analgesics were used when determined appropriate by veterinary medical staff.

### Human subjects

All HIV-infected subjects were antiretroviral therapy (ART) naïve or had not taken antiretrovirals for ≥ 6 months prior to blood donation. The median viral load for this HIV-infected cohort was 48,977 copies/ml (range 700–162,200). The median CD4+ T cell count was 471 cells/ul of blood (range: 4–838). There were no significant differences in sex or age between HIV-infected donors and uninfected controls. Exclusion criteria for all human subjects included having ever received the BCG vaccine or having a previous Mycobacterial infection; all donors were verified to be PPD-negative.

### Sooty mangabeys

Sooty mangabey blood was either obtained from uninfected animals or those that were naturally SIV infected while being group-housed with SIV+ mangabeys at the Yerkes National Primate Research Center. There were no differences in weight, age, or CD4+ T cell count between the SIV-infected and uninfected mangabeys. The median viral load for the SIV+ mangabeys was 97,723 copies/ml of plasma (range: 25,704–218,776).

### *Mycobacteria* culture and preparation

*M*. *bovis* BCG (Russia) was grown in 7H9+GAT media at 37°C with constant rolling for at least two doublings. Upon reaching log growth phase (OD_600_ 0.3–0.7), the bacteria were washed twice in 1x PBS and resuspended in complete RPMI (RPMI1640+10% fetal bovine serum, FBS) in the absence of antibiotic. The volume added to each sample was calculated via the OD_600_ of the resuspended sample for each experiment, assuming OD_600_ of 1 = 4.5x10^8^ colony forming units/ml.

#### PBMC extraction and BCG exposure for Nanostring

PBMC extracted from a Ficoll gradient were resuspended in RPMI+10%FBS. One million cells were plated in triplicate in 96-well flat-bottom plate and rested 1h at 37°C+5% CO_2_. 1x10^6^ CFU BCG (RPMI+10% FBS; enumerated via OD_600_) was then added in 100ul, bringing all final volumes to 200ul. PBMC were incubated with BCG for 4 hours at 37°C with 5% CO_2_. For all the experiments described below, we chose to use the term “exposure” when referring to PBMC or whole blood incubation with BCG, as the cells could have been stimulated through pathogen-associated molecular patterns (PAMPs) or through infection/engulfment of the bacteria. Following incubation, cells from triplicate wells were pooled and pelleted. 100ul lysis buffer (RLT; Qiagen with Beta-mercaptoethanol), was used to wash/lyse remaining (adherent) cells in the plates before adding it to the cell pellet. All disrupted cell pellets were stored at -80°C until RNA extraction.

### Measurement of Gene Expression Profile

mRNA was isolated from BCG-exposed PBMC using the Qiagen RNeasy kit according to the manufacturer’s protocol. Following isolation, mRNA was quantified and quality checked by measuring the A_260/280_ and A_260/230_ ratios prior to analysis. Probes specific for 248 target genes related to cytokine and chemokine signaling, TLR signaling, antigen processing, and NK cell-specific markers were designed and manufactured by Nanostring Technologies, and analyzed using the Nanostring nCounter analysis system as previously described [25, 26]. Probes were designed to target the human gene target sequence, but unique probes were generated for targets in which significant homology was not observed between humans and a macaque genome (used in lieu of having sooty mangabey sequences, as the whole sooty mangabey genome sequence was not publicly accessible at the time the Nanostring probeset was generated). mRNA samples were incubated overnight at 65°C prior to loading onto the Nanostring prep station to remove unbound probes. Probes were then utilized for reading on the Nanostring digital analyzer. Each sample was run with 6 internal positive controls and 8 internal negative controls.

### Nanostring Data Analysis

Nanostring gene expression data were normalized using the designated internal controls GAPDH, ALAS1, and HPRT. The arithmetic mean of the negative control conditions plus 2 standard deviations of the mean (which totaled 35 copies for the humans and 17 copies for the mangabeys) was used as a cutoff for gene expression for each species; Genes with ≥50% of the samples below the negative cutoff were excluded from future analyses. Differences in baseline gene expression (unstimulated controls) between HIVneg and HIV+ donors or SIVneg and SIV+ mangabeys were identified via a Mann Whitney non-parametric test. One caveat to the assessment of unsorted immune cells is that differences in the distribution of cells present within PBMC may impact the types/numbers of transcripts that are present. To control for this, all stimulated samples had a paired unstimulated control sample, which permitted an assessment of the baseline differences in our different groups that may affect responses in addition to BCG. As such, differentially expressed genes (DEGs) in HIVneg and HIV+ donors as well as SIVneg and SIV+ mangabeys were determined through a paired t-test (unstimulated vs. stimulated for each subject) following BCG exposure. For all analyses, a false discovery rate (FDR) threshold of 10% was used to control for multiple comparisons through Benjamini–Hochberg methodology [27]. Additionally, for the effect of BCG exposure, we used an absolute fold change threshold of >1.5. The Kyoto Encyclopedia of Genes and Genomes (KEGG) pathway enrichment analysis (from WebGestalt, Web-based Gene SeT AnaLysis Toolkit) was used to identify pathways significantly enriched in genes differentially expressed between groups at baseline [28]. Using this method, the hypergeometric test was used to calculate the statistic for enrichment of each pathway within regulated genes and the Nanostring gene panel serving as the reference gene set. The p-value was adjusted by Benjamini–Hochberg (BH) methodology, and KEGG pathways with adjusted p<0.05 were considered to be enriched. All Nanostring gene expression data has been uploaded to the open source NCBI Gene Expression Omnibus (GEO) website (Accession Number: GSE81926).

### Whole blood BCG exposure

Whole, herparinized blood (100ul) from HIVneg and HIV+ human donors or SIVneg and SIV+ mangabeys was dispensed into 5ml round-bottom polypropylene FACS tubes. Bacteria were added based on the average number of leukocytes in 100ul/whole blood: 5x10^3^ (multiplicity of infection or MOI 0.5), 10^4^ (MOI 1), 5x10^4^ (MOI 5), or 10^5^ (MOI 10) bacteria /ul whole blood were added for initial dose-response experiments, while 10^4^ (MOI 1) or 10^5^ (MOI 10) was used for subsequent experiments. Data depicted in the figures within the manuscript utilized an MOI of 10 unless otherwise indicated. The incubations of peripheral blood cells and BCG were placed at 37°C + 5%CO_2_ for 1 hour before Brefeldin A (0.2ug/ml) was added, and after 5 additional hours (6 hours total) placed at 4°C overnight. Each experiment included a negative (unstimulated) control.

### Intracellular cytokine staining

BCG stimulated whole blood cells were washed once in 1x PBS+2%FBS before extracellular antibodies were added: CD3 APC-Cy7 (clone SP34-2) (alternatively CD3 FITC was also used), CD14 PE-Cy7 (clone M5E2), or Live dead Aqua (1ul, Invitrogen). Samples were stained for a total of 30 minutes on ice and washed in 1x PBS+2%FBS. Samples were then resuspended in residual wash buffer before 750ul FACS juice (25% BD Bioscience FACS lyse + 0.05% Tween-20 in dH_2_O) was added for 10 minutes with occasional vortexing. The samples were then washed twice in 1x PBS+2%FBS before the TNF-α (APC, clone MAb11) and IL-12 (PE, clone C8.6, Miltenyi Biotec) antibodies were added to conduct the intracellular cytokine staining. After a 30 minute-incubation on ice, and final wash in 1x PBS+2%FBS, the cells were resuspended in 100ul 2% paraformaldehyde prior to analysis on a BD LSRII using FACSDiva software. All antibodies listed are from BD Bioscience unless otherwise stated. The fluorescence minus one (FMO) control data has been included for the IL-12 and TNF-alpha staining such that the positive cellular populations can be clearly identified (S1 Fig). The monocyte population is identified as CD3 negative, CD14 bright and Side Scatter-mid. Using this combination of cellular lineage markers together with the unique side-scatter profile of monocytes has minimized potential for B cell or DC contamination.

### IFN-γ ELISA

Supernatants from whole PBMC (matched samples from the Nanostring gene expression analyses described above) that were stimulated with BCG for either 4h or 18h were collected and stored at -80C until use. The levels of IFN-gamma protein was not detectable after 4 hours (not shown), and therefore the 18 hour data was evaluated here (both undiluted and at a 1:10 dilution). All samples were thawed on ice and assayed in duplicate. The IFN-γ ELISA (ThermoFisher) was performed according to the manufacturer’s protocol. Optical densities at 450nm and 540nm were measured, and the concentration of protein in each sample was calculated by averaging the optical density of the duplicate samples and plotting against a standard curve.

### Data analysis

All flow data were analyzed using FlowJo version 8.8.6. Statistical analyses were performed using GraphPad Prism version 5 and the R project [29]

## Results

### Monocytes from HIV-infected subjects exhibit reduced IL-12 production in response to BCG

Monocytes are important components of the innate response for controlling mycobacterial infections. Alterations in monocyte functional responses to mycobacteria have been observed in HIV-infected persons [30–32], and this likely contributes to the increased susceptibility to mycobacterial diseases. To evaluate proinflammatory cytokine production by monocytes from HIV-infected and control subjects, whole blood was exposed to *M*. *bovis* BCG (BCG) for 6 hours prior to intracellular cytokine staining and multiparameter flow cytometry. Following 6 hours, human monocytes exhibited a dose-dependent response in the proportion of TNF-α+ monocytes following addition of BCG at MOIs 0.5, 1, 5 and 10 (S2 Fig). These dose titration experiments determined that MOI of 1 was the smallest dose reproducibly yielding monocyte TNF-α production above background, and MOI of 10 identified a robust cytokine response, without an increase in cell death following a 6-hour exposure. Thus, an MOI of 10 was utilized for the studies described here (unless noted otherwise).

**Fig 1:**
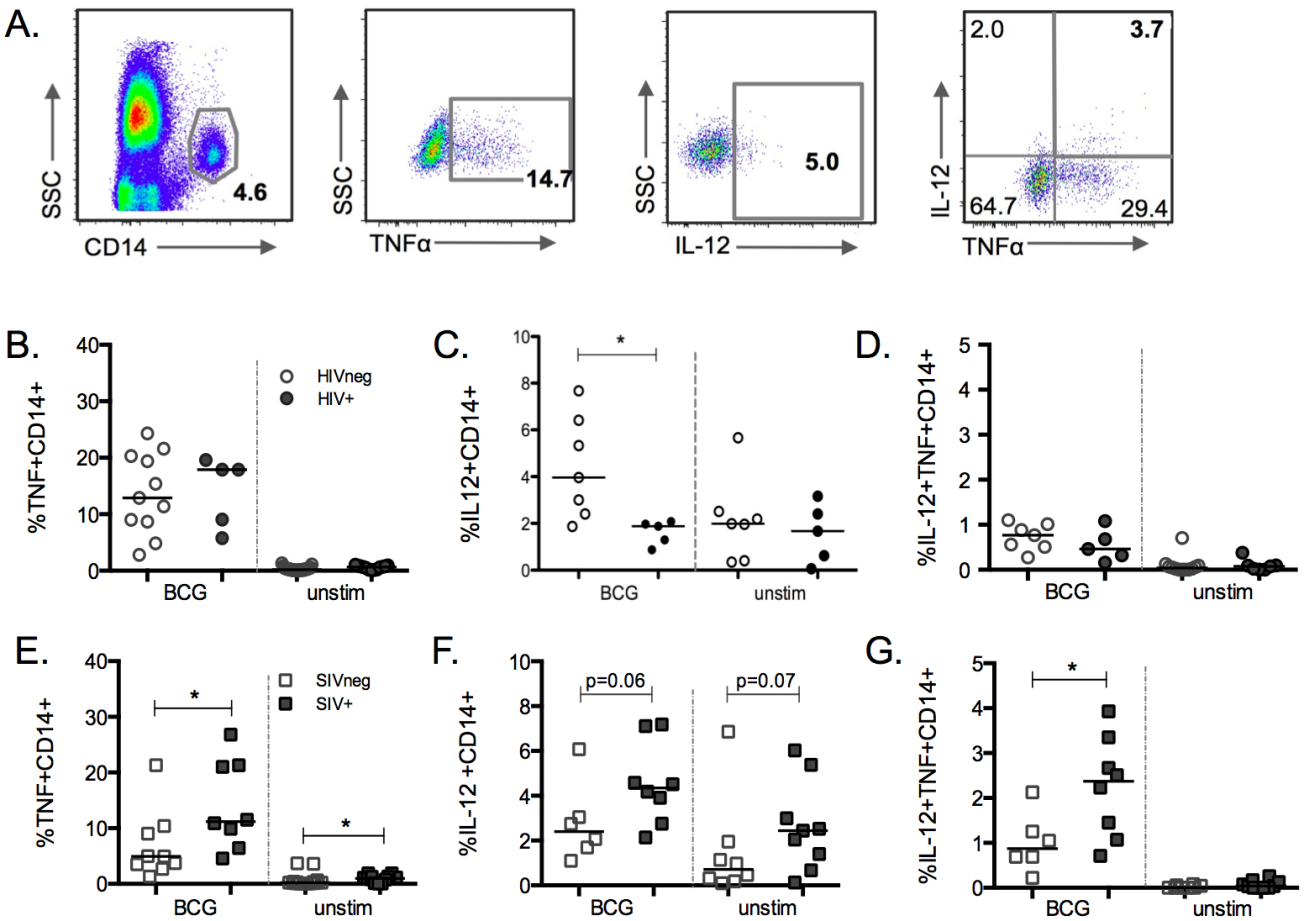
Whole blood monocyte proinflammatory cytokine production in HIVneg and HIV+ donors or SIVneg and SIV+ mangabeys following exposure to *M*. *bovis* BCG. (A) Representative flow cytometry plots and gating strategy of cytokine-producing monocytes, which were first defined as being live and CD3 negative (not shown), CD14+, and producing TNF-α, IL-12, or both following 6h exposure to BCG. (B-G) Percentage of TNF-α+ (B, E), IL-12+ (C, F), or double-positive (TNF-α+ and IL-12+) monocytes (D, G) in HIVneg (unfilled circles) and ART-naive HIV+ (filled circles) humans or SIVneg (unfilled squares) and SIV+ (filled squares) mangabeys following ex vivo BCG exposure. Lines represent the medians for each group (*p<0.05, Mann-Whitney).

CD3-CD14+ monocytes (Fig 1A) from HIV-negative (HIVneg) or HIV+ donors were identified via flow cytometry, and were assessed for expression of TNF-α (Fig 1B), IL-12 (Fig 1C) or both TNF-α and IL-12 (Fig 1D). A similar percentage of TNF-α-producing monocytes were observed in response to BCG between HIVneg and HIV+ donors at MOI 10 (Fig 1B). In contrast, HIV+ donors exhibited a lower proportion of IL-12 producing monocytes (median = 1.9%) compared to uninfected subjects (median = 4%; p = 0.017; Fig 1C) following BCG exposure. Finally, following BCG exposure the monocytes from HIV+ subjects displayed a similar proportion of TNF-α and IL-12 double positive monocytes compared to uninfected donors (Fig 1D). Together, these findings indicate that HIV infection is associated with a monocyte-specific lack of IL-12 upregulation in response to BCG. This lack of monocyte IL-12 upregulation observed was unique to BCG, as it was not observed following LPS stimulation (S3 Fig).

### Monocytes from SIV-infected mangabeys exhibit increased cytokine production in response to BCG

The non-pathogenic SIV infection observed in sooty mangabeys provides an important model for comparison to pathogenic HIV infection following a BCG exposure. Similar to humans, the sooty mangabey whole blood monocyte response to BCG also resulted in a proportional increase in cytokine response with increasing MOI of BCG (S2 Fig). However, mangabey monocyte BCG response did differ slightly, as a peak response was elicited from the monocytes when an MOI of 5 was used (S2 Fig), compared to an MOI of 10 for the humans (the dose utilized for all of the experiments described herein).

Following BCG exposure, SIV+ sooty mangabeys displayed an increase in the frequency of TNF-α-positive monocytes at MOI 10 (median = 11.2%; Fig 1E) compared to SIVneg mangabeys (median = 4.9; p = 0.03) as well as at an MOI of 1 (SIV+ 2.1, SIVneg 1.1; p = 0.02, data not shown). SIV+ mangabeys also displayed a clear trend toward an increased proportion of IL-12+ monocytes in response to BCG at MOI 10, although this did not reach statistical significance (p = 0.059). This trend was also observed in the unstimulated cells (Fig 1F, right side), with SIV+ mangabeys producing more IL-12 basally (median = 2.5%) compared to SIVneg mangabeys (median = 0.72%; p = 0.08). Further, in contrast to HIV+ humans, SIV+ mangabeys significantly upregulated monocyte IL-12 production in response to BCG (Fig 1F). Finally, SIV+ mangabeys displayed an increased percentage of TNF-α/IL-12 double positive monocytes (median = 2.4%) compared to SIVneg mangabeys (median = 0.87%; p = 0.024) following BCG exposure (Fig 1G). Together, these findings indicate that SIV infection of mangabeys was associated with a more robust BCG-associated production of inflammatory cytokines from monocytes when compared to their SIVneg counterparts.

### Transcriptomic assessment of PBMC from HIV-infected subjects following BCG exposure

**Fig 2:**
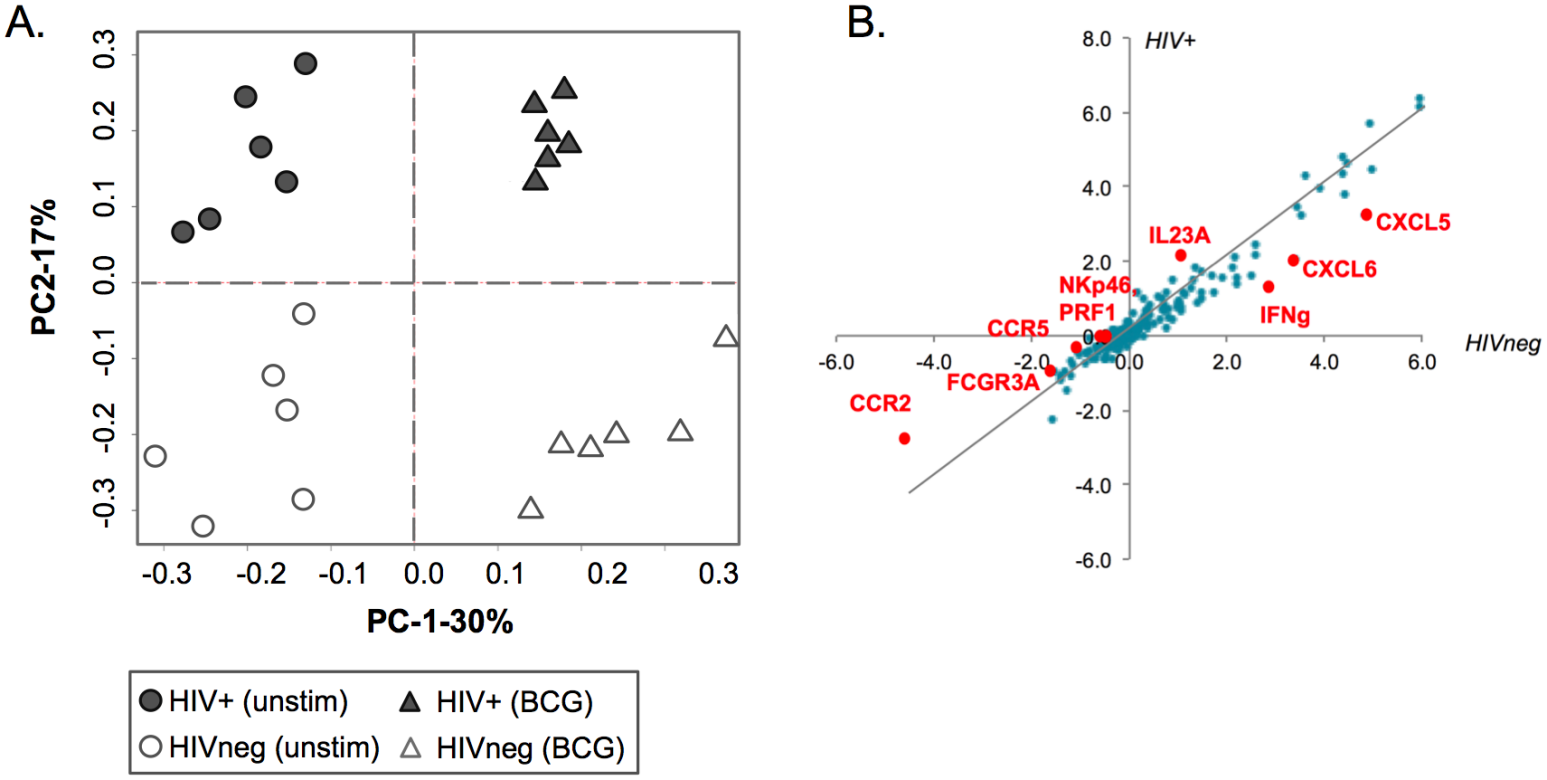
Gene expression analysis of HIVneg and HIV+ PBMC following 4 hour BCG exposure. (A) Semi-supervised hierarchical clustering via Principal Component Analysis (PCA) of 199 immunological gene targets (chosen *a priori*) was performed on RNA expression from whole PBMC following 4h exposure of BCG and controls (without BCG), in ART-naïve HIV+ or HIVneg donors. PC-1 (x-axis) represents the component responsible for the largest variation in the data set and explains 30% of the variation while PC-2 (y-axis) represents the component contributing the second highest degree of variability to the dataset, explaining 17% of data variation. (B) Fold change in expression these genes from HIVneg (x-axis) or HIV+ (y-axis) donors after 4h BCG exposure are shown via scatterplot. Genes that diverge significantly from the trendline (with slope = 1) are differentially regulated between HIVneg and HIV+ donors following BCG exposure and are denoted in red.

Transcriptomic analysis permits a broad-scale evaluation of gene expression. Using the Nanostring platform, we evaluated the expression of 248 gene targets from whole PBMC following 4 hours of BCG exposure of 6 ART-naïve HIV+ donors and 6 HIVneg controls. A semi-supervised hierarchical clustering via principal component analysis (PCA) was undertaken to identify the key factors that were influencing the gene expression and sample clustering in our data set. The effects of BCG exposure are captured by the highest principal component (Fig 2A; PC-1, x-axis), indicating that exposure to BCG influenced the PBMC transcriptome profile to the greatest extent. HIV status was the second most influential factor affecting gene expression, as observed by distinct clustering of HIVneg and HIV+ donors in both the BCG stimulated and unstimulated controls (Fig 2A; PC-2, y-axis).

The PCA demonstrates that transcriptomic differences can be observed between the HIV+ and uninfected subjects in the absence of BCG exposure (Fig 2A, comparing filled to open circles). Because these baseline (pre-exposure) immune differences may influence the overall response to BCG, we evaluated them in further detail. After correcting for multiple comparisons (FDR <10%), we determined that 29 genes (~12% of the total gene set) were differentially expressed at baseline between HIVneg and HIV+ donors. KEGG analysis of genes that were either upregulated in HIV+ donors (22 genes) or downregulated (7 genes) compared to the uninfected controls identified that upregulated genes significantly enrich the NK cell-mediated cytotoxicity pathway (adjusted p<0.001) (S1 Table). As expected, genes associated with immune activation, such as cell proliferation marker Ki67 (MKI67) and RANTES (CCL5) were also upregulated in HIV+ donors (S1 Table, S4 Fig). The seven genes expressed to a lower extent in HIV+ donors were primarily associated with cytokine-cytokine receptor pathways.

**Fig 3:**
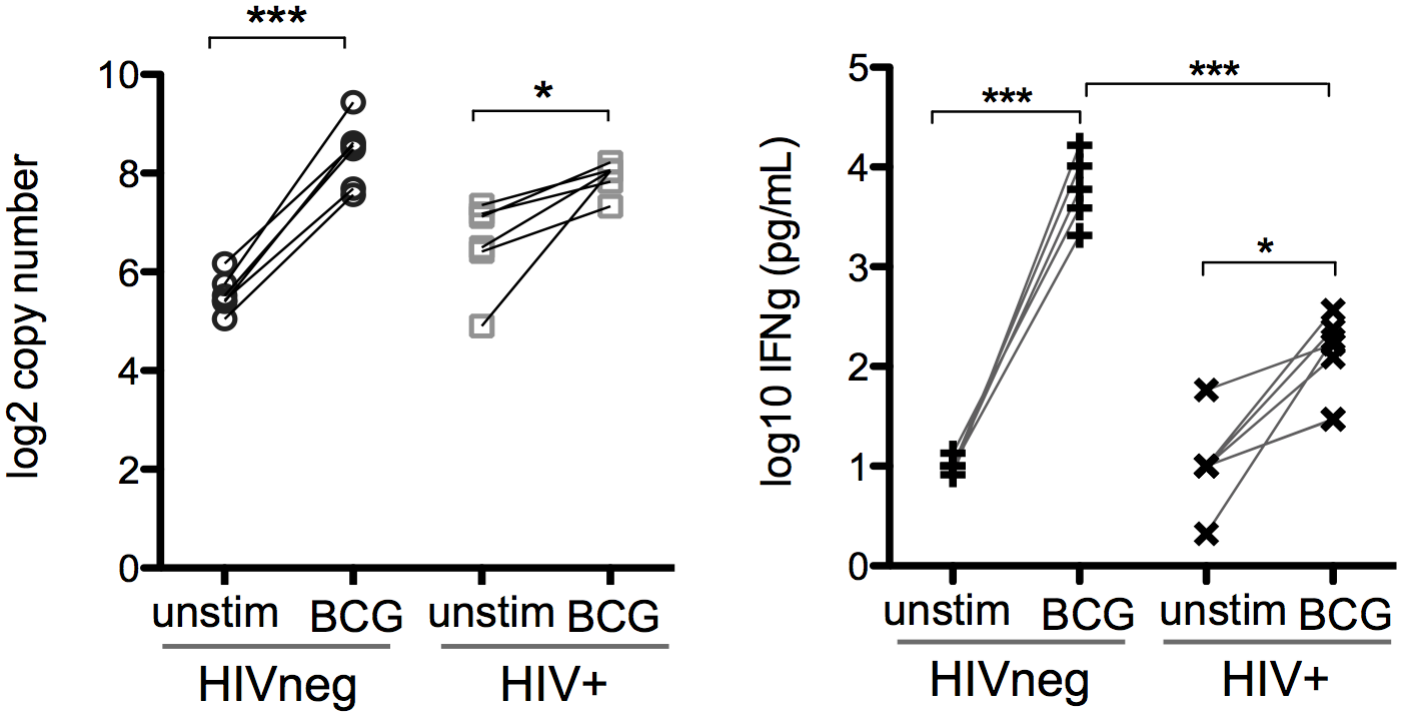
HIV infection is associated with a deficiency in IFNg mRNA and protein upregulation in response to M. bovis BCG. mRNA expression of IFN-γ in response to 4h of BCG exposure from HIVneg or ART-naïve HIV+ PBMC was measured via Nanostring analysis (left panel; HIVneg p<0.0001, HIV+ p = 0.02). IFN-γ protein was measured in supernatants from matched PBMC stimulations at an 18h time point (right panel; HIVneg-unstim vs HIVneg BCG p = 0.0001; HIV+ unstim vs HIV+ BCG p = 0.004; HIVneg-BCG vs HIV+ BCG p<0.0001; HIVneg unstim vs. HIV+ BCG n.s.). Statistical evaluation was performed via a two-tailed t-test on log-transformed data.

**Fig 4:**
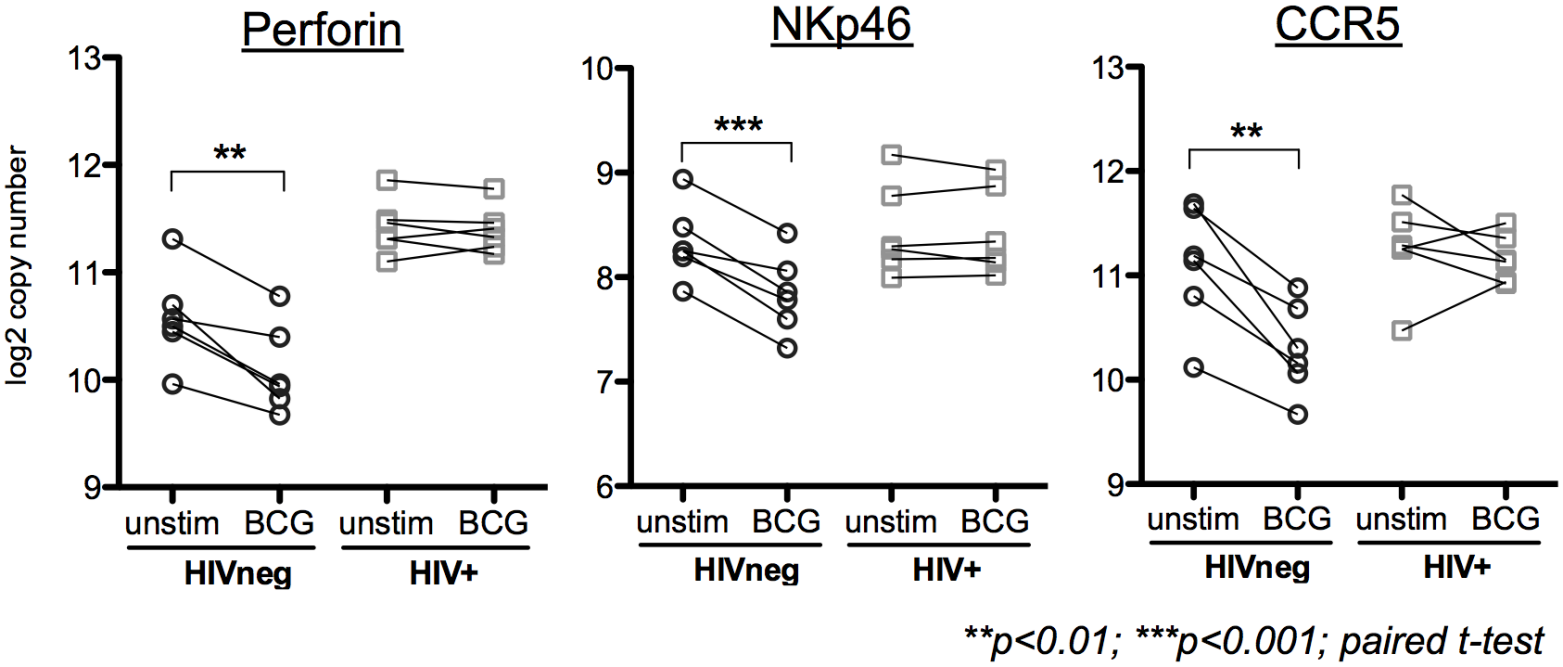
Selected genes that demonstrate HIV status-specific effects of BCG exposure. Fold change in the expression of 199 immunologic genes in response to 4h BCG exposure of PBMC from HIVneg (circles) or ART-naïve HIV+ (squares) was assessed via Nanostring gene expression analysis. Twelve genes remained significant following correction for multiple comparisons at an FDR threshold <10% (listed in Table 1). Following the identification of these genes, further validation of the differences in response between HIVneg and HIV+ subjects was identified via a paired t-test: PRF1 (HIVneg p = 0.0045, HIV+ p = n.s), and NKp46 (HIVneg p = 0.0008, HIV+ p = n.s) perform cellular effector functions, and CCR5 (HIVneg p = 0.0029, HIV+ p = n.s) is an HIV coreceptor.

To identify transcriptomic differences between HIVneg and HIV+ subjects in response to BCG exposure, the samples were evaluated in a pairwise fashion prior to and following BCG exposure. Differentially-expressed genes (DEGs) for each of the 4 groups were classified as having an absolute fold change >1.5 (FDR <10%). Fold changes of gene expression in HIVneg or HIV+ donors following BCG exposure for all genes expressed above the negative cutoff are displayed via scatterplot (Fig 2B). Overall, the PBMC response to BCG was similar between HIVneg and HIV+ donors. However, some key differences were observed, including 12 genes that displayed a BCG specific effect between HIV-infected and uninfected donors at a false-discovery rate of <10% (Fig 2B and Table 1). Following the identification of these genes, further validation of the differences in response between HIVneg and HIV+ subjects was identified via a paired t-test. IFN-γ (fold change 7.2 in HIVneg donors, p<0.0001 compared to fold change 2.5 in HIV+ donors, p = 0.02, Fig 3) is important for cellular-mediated anti-mycobacterial responses. The lower IFN-γ induction by HIV+ donors indicates an impaired early IFN-γ response to BCG compared to healthy donors (Fig 3A). This finding observed at the mRNA levels was also confirmed by testing the levels of secreted IFN-γ in stored supernatants (following 18h of stimulation) from the PBMC stimulations (Fig 3B). Other genes displaying significance include NKp46, perforin and CCR5, which were downregulated only in HIVneg subjects following BCG exposure (Fig 4). Therefore, these gene transcripts, which are normally downregulated in healthy individuals, maintain pre-BCG-exposure levels in the HIV+ donors. Together, the differential expression of IFN-γ, NKp46, perforin and CCR5 expression demonstrate an altered expression of effector cells (NK and T cells), and suggest a potential role for altered effector responses to BCG in HIV+ donors.

**Table 1:** Genes associated with HIV-status-specific effects following BCG exposure ex vivo.

Gene	p-value	FDR
**IFNg**	**<0.001**	**0.049**
CCR2	0.001	0.079
CCR5	0.002	0.079
CXCL6	0.002	0.079
CXCL5	0.003	0.079
RORC	0.003	0.079
FCGR3A	0.003	0.079
PRF1	0.003	0.079
NKp46	0.004	0.079
IL23A	0.004	0.079
BATF	0.004	0.079
TLR4	0.005	0.084

### Transcriptomic assessment of PBMC from SIV-infected mangabeys following BCG exposure

**Fig 5:**
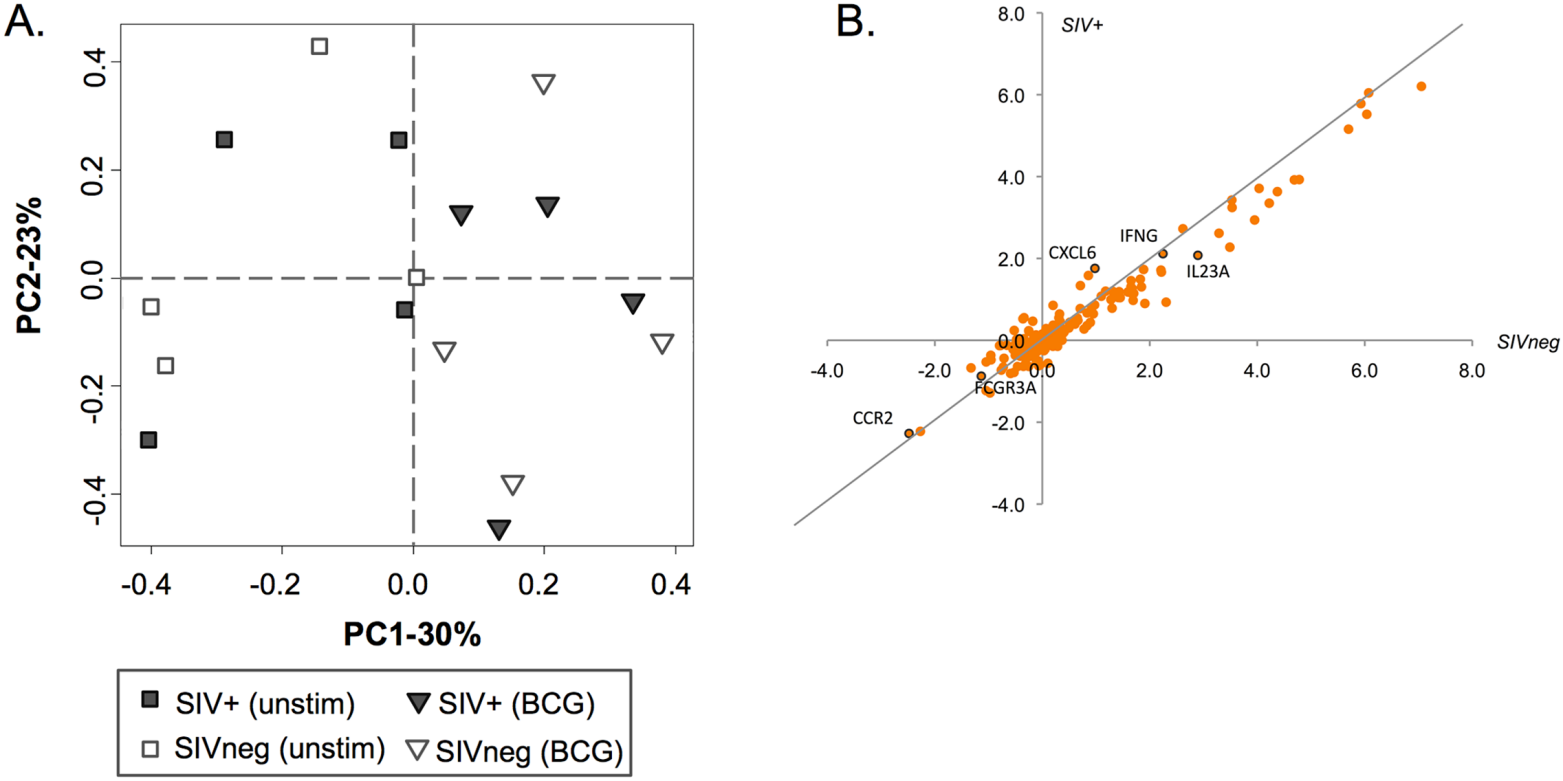
Gene expression analysis of SIVneg and SIV+ mangabey PBMC following 4 hour BCG exposure. Semi-supervised hierarchical clustering via Principal Component Analysis (PCA) of 199 immunological gene targets (chosen *a priori*) was performed on RNA from whole PBMC following 4h BCG exposure and controls (without BCG), in SIV+ or SIVneg mangabeys via PCA (A). PC-1 (x-axis) represents the component responsible for the largest variation in the data set and explains 30% of the variation, while PC-2 (y-axis) represents the component contributing the second highest degree of variability in the dataset of 23%. Fold change in expression of these genes in SIVneg (x-axis) or SIV+ (y-axis) subjects after BCG exposure is depicted via scatterplot (B). No genes were found to diverge significantly from the trendline. A black outline denotes the genes that displayed statistically significant differences between HIVneg and HIV+ donors, for comparison.

A Nanostring transcriptomic analysis was also undertaken using PBMC from 4 SIV+ and 4 SIVneg (control) sooty mangabeys following BCG exposure as described above for human PBMC. Of the 248 targets selected *a priori*, we analyzed in detail 199 genes that were expressed above background in ≥50% of mangabey samples (analyses were done on these 199 genes). Principal component analysis of 4 SIVneg and 4 SIV+ sooty mangabeys demonstrated that BCG exposure was captured by the first principal component (PC1-30%; x-axis; Fig 5A), similar to what was observed in humans (Fig 2A). However, SIV status did not contribute significantly to sample clustering before or after exposure, in contrast to what was observed for humans. Upon further investigation, we determined that SIV status was captured by the 8^th^ component of gene clustering, suggesting that contribution of SIV infection to overall gene expression variation in sooty mangabeys is minor compared to that induced by BCG exposure and compared to the effect that HIV exerts on gene expression in humans. Assessment of gene expression in the mangabeys did not reveal any significant differences between the SIV-infected and SIVneg mangabeys at baseline as depicted by scatterplot (Fig 5B), further supporting the observation from the PCA. In contrast to what was observed in HIV+ donors, we found no evidence that SIV status affects the quality or magnitude of the immune response to BCG. This was in accordance with our hypothesis, as the samples were taken during the chronic phase of the SIV infection, when SIV-induced systemic changes are generally not observed in mangabeys [9].

### Comparison of human and mangabey immune responses to BCG

**Fig 6:**
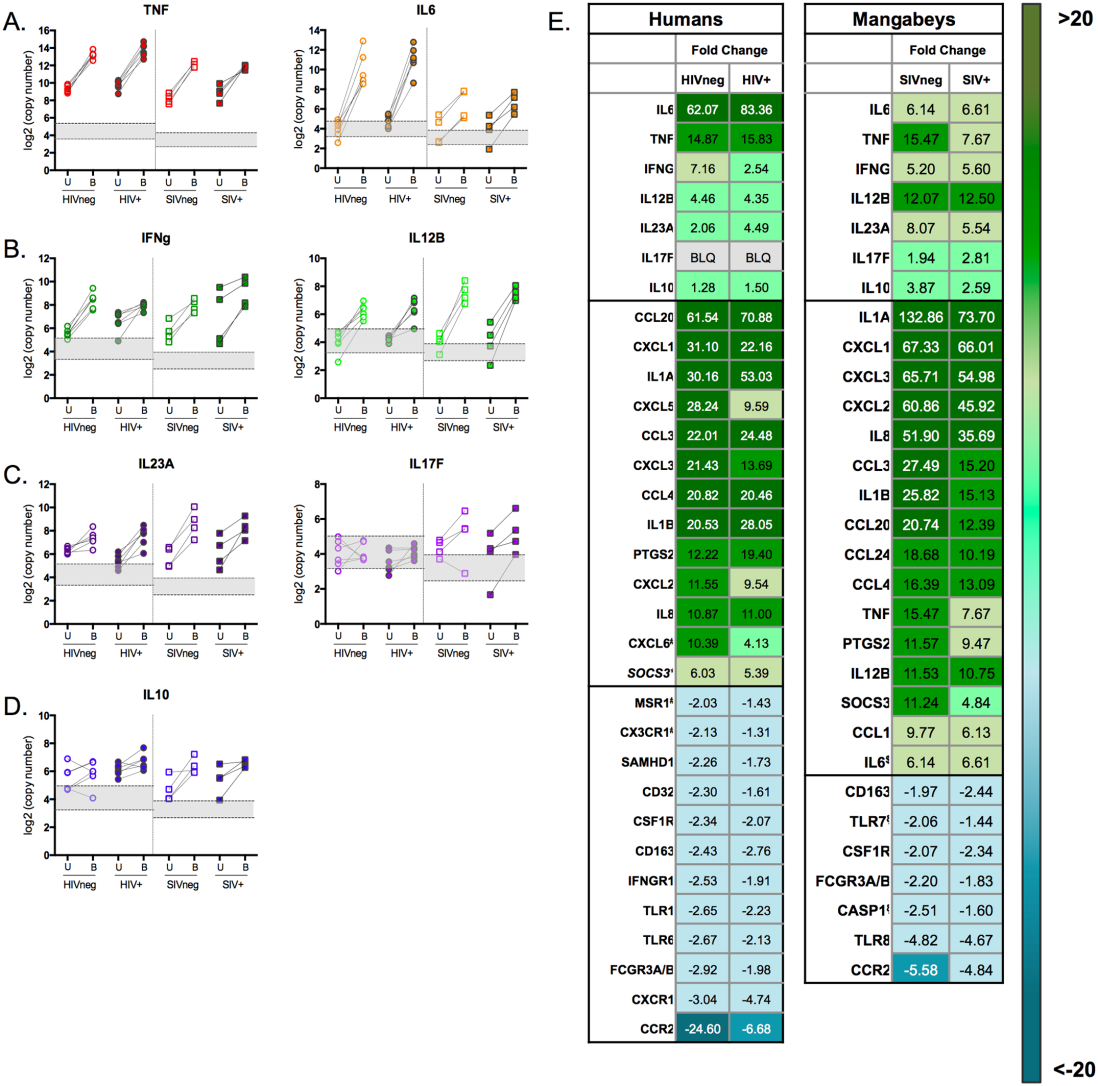
Whole PBMC cytokine gene regulation in HIVneg or HIV+ donors following BCG exposure. Plots demonstrate regulation of key proinflammatory. (A), Th1 (B), Th17 (C) and immunoregulatory genes (D) following 4h BCG exposure. Log2 copy number of RNA in an unstimulated control (U) or matched BCG-stimulated (B) samples in either humans (circles) or mangabeys (squares). The lower bound of the gray shaded portion of the graph represents the mean of the negative controls, and the upper bound represents two standard deviations above the mean, which ultimately determined the cutoff for expression. The most highly up- and down-regulated genes in humans or mangabeys are displayed via a heat map (E). FC: fold change; BD: below detection.

A proper balance of inflammation and immunoregulatory responses is critical to cell-mediated immunity that promotes killing of intracellular pathogens, like mycobacteria, while limiting tissue damage. We were therefore interested in the expression of key genes known to be important in the response to mycobacteria, including genes from the proinflammatory (TNF-α, IL-6), Th1 (IL-12, IFN-γ), Th17 (IL-17, IL-23), and immunoregulatory (IL-10) cytokine families (Fig 6) during HIV or SIV infections, many of which play roles in induction or suppression of inflammation. We found that TNF-α mRNA was upregulated equally in humans regardless of HIV status (fold change of 14.9 for HIVneg vs 15.8 for HIV+). It was upregulated to a similar extent in SIVneg mangabeys, but to about half that level in SIV+ mangabeys (fold change of 15.5 for SIVneg and 7.7 for SIV+). IL-6 dominated the overall response in humans (upregulated 62- (HIVneg) or 83- (HIV+) fold compared to unstimulated controls. In contrast, the IL-6 response was relatively attenuated in sooty mangabeys (6.2-fold upregulation in both SIVneg and SIV+ mangabeys), which instead demonstrated a transcriptional response dominated by IL-1A transcripts (Fig 6E). We also observed a decrease in IFN-γ upregulation by HIV+ donors compared to HIVneg (2.5-fold vs 7.2-fold), while no SIV-dependent difference was noted in the ability of sooty mangabeys to upregulate IFN-γ (Fig 6B), which was upregulated to a similar extent to that observed in HIVneg humans. Overall, mangabeys exhibited more robust IL-12 production following BCG exposure (fold change of 4.4 for humans vs. 12 for mangabeys), as well as more IL-23 and IL-17 (Fig 6E), and IL-10 (Fig 6D).

## Discussion

**Fig 7:**
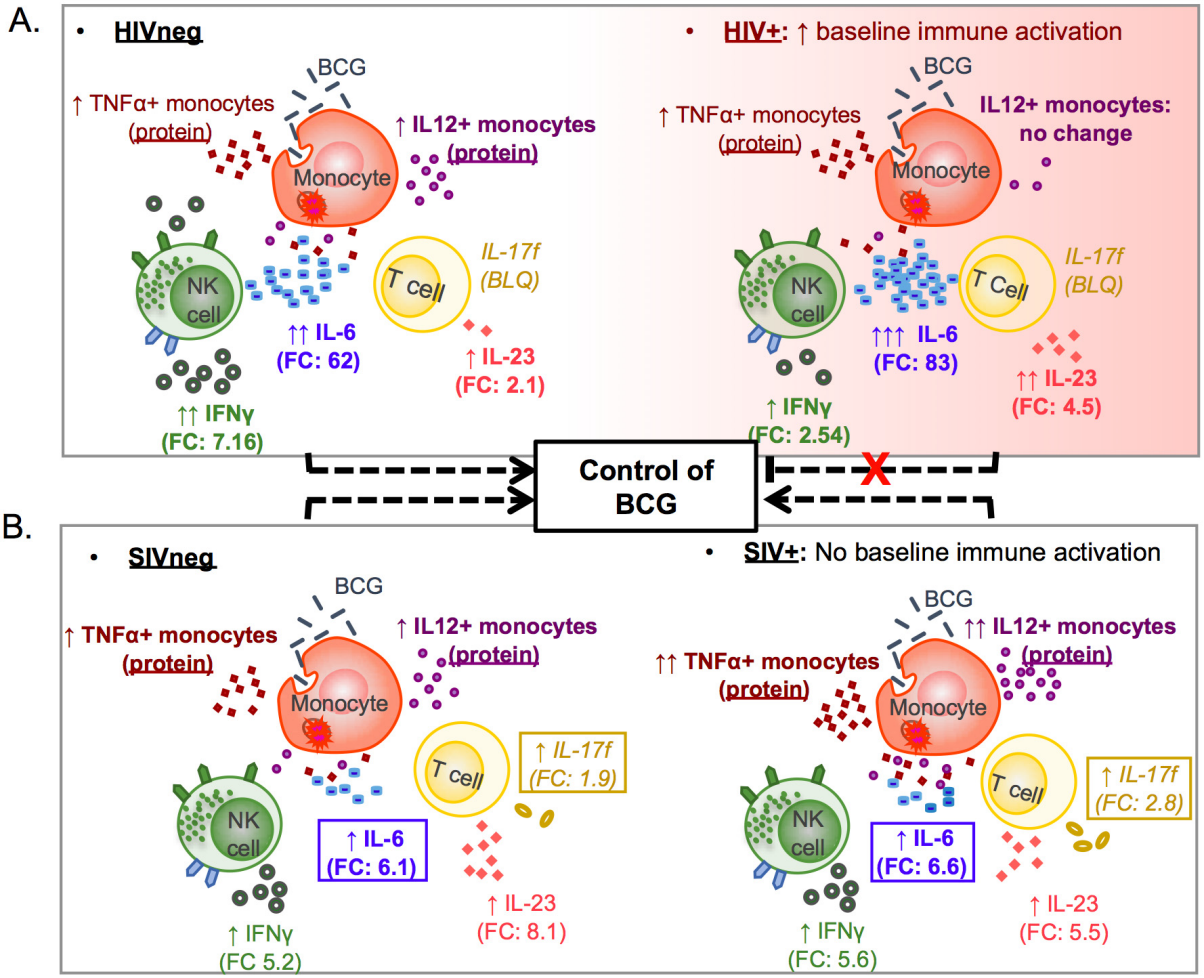
Model of differential cellular responses during the innate immune response to BCG during pathogenic HIV or non-pathogenic SIV infection. Cytokines that are differentially expressed between HIVneg and HIV+ humans (A) or SIVneg and SIV+ mangabeys (B) are presented without boxes and are in bold type font. Arrow(s) represent the up or down modulation of these immune modulators in response to BCG, and fold change (FC) is presented as numeric values. Cytokines expressed differently between human subjects and mangabeys are boxed (B).

Comparing and contrasting immune responses between pathogenic (HIV-humans) and nonpathogenic (SIV-mangabeys) lentiviral infections has previously provided key insights into the differential factors that impact the clinical outcome and AIDS progression [10, 33–35]. Our goal here was to determine how these disparate disease courses impact the immune response profile to BCG, the live-attenuated vaccine administered to mitigate tuberculosis-associated disease, that also has the potential to become an opportunistic pathogen in immunocompromised hosts. To simplify the intra and inter-species comparisons of the transcriptomic and flow cytometric findings associated with BCG exposure, we generated a model to highlight some of the key immune modulators and cell types involved in this response (Fig 7). The model includes three different immune cell types with potential to play important roles in the BCG-specific early innate response: monocytes, NK cells, and T cells. We do not rule out a role for other immune cell populations (different T cell subsets or B cells, for example) present in the peripheral blood compartment to contribute to the observed data.

Our first objective was to measure whole blood monocyte expression of TNF-α and IL-12 following BCG exposure. We chose to use whole blood for these *ex-vivo* analyses, which has the benefit of providing monocytes with the most physiologically relevant environment for responding to the bacteria. Through these analyses, we observed an HIV-associated monocyte-specific lack of IL-12 upregulation in response to BCG, resulting in a significantly lower IL-12 production compared to uninfected donors (Fig 6A, left side). Previous findings have identified an HIV-associated reduction in IL-12 production by alveolar macrophages [36] and PBMC [37] in response to *Staphylococcus aureus*. Peripheral blood monocytes [38] as well as macrophages and myeloid-derived dendritic cells also exhibit decreased IL-12 production during HIV or pathogenic SIV infections [39]. Our findings here with *M*. *bovis* BCG are in agreement with these previous studies [36–39]. Assessment of gene expression following BCG exposure did not identify any difference in the expression of IL-12 in HIV-infected subjects, in contrast to our flow cytometric assessment of IL-12 protein levels. This discrepancy could potentially be due to post-transcriptional regulation of IL-12, which has been observed previously [40] or through an obscured ability to detect cell-specific differences in IL-12 mRNA levels in bulk PBMC.

HIV-infection status resulted in distinct subject clustering due to genes that were differentially regulated in the HIV-infected compared to the uninfected control subjects, even prior to BCG exposure (S2 Fig). The majority of these genes were more highly expressed in HIV-infected donors and represented genes predicted to be upregulated during chronic viral infection, including those associated with cellular activation (Ki67, FAS, HLA-A, IL-12RB1), and NK cell activation (NKG2A, also Perforin, Granzyme B). Following exposure to BCG, PBMC from HIV+ subjects upregulate IFN-γ mRNA and protein, but not to the level observed in healthy donors. This reduced IFN-γ response may alter the intracellular killing capacity and the clearance of mycobacteria, and may lead to reduced activation of other effector cells, like monocytes. Major known sources of IFN-γ include NK cells and CD8+ T cells. Indeed, impaired Mtb-specific CD8 T cell effector functions, such as degranulation and proliferation have been observed in previous studies [41]. However, together with differentially regulated levels of NKp46 and perforin following short-term BCG exposure, the lack of IFN-γ upregulation suggests redundancy in a dysregulated NK cell response to BCG in HIV+ donors. These data support previous studies that demonstrate NK cells play a role in the innate response to mycobacteria [42–45], become dysfunctional during HIV infection, and warrants future exploration.

Finally, we observed that following BCG exposure IL-23 mRNA was upregulated more than double in HIV+ donors compared to uninfected controls (Figs 6 and 7; including average fold changes of 2.06 for HIVneg and 4.49 for HIV+). Overall, our data are consistent with a recent study that shows an HIV gp120-mediated increase in IL-23 production from myeloid cells following stimulation with LPS, which was then linked to a SOCS1-induced decrease in Th1 cytokines IL-12 and IFN-γ [46]. IL-23, together with IL-12 and IFN-γ, has also been identified as important for an effective response to mycobacteria in mice [47], and in healthy persons (reviewed in [48]) by helping to bridge innate and adaptive immunity. Therefore, the observed disruption in the IL-23/IL-12/IFN-γ signaling may contribute to the decreased ability for HIV+ donors to mount an effective response against mycobacteria.

In contrast to the decreased percentage of IL-12+ monocytes in HIV+ donors, we observed an increase in the whole blood monocyte cytokine response to BCG exposure in SIV+ compared to uninfected mangabeys. These data further support that SIV+ mangabeys are able to mount a robust pathogen-mediated activation response despite the more global suppression of inflammation that results in a lack of chronic immune activation [9, 49], reviewed in [50, 51]. As observed in humans, we found a BCG-associated upregulation of both TNF-α and IL-12 transcripts in all mangabeys analyzed, regardless of infection status (Fig 7).

In addition, we observed no differences in baseline markers of effector molecules associated with immune activation in SIV+ sooty mangabeys and no significant differences in response to BCG between SIV+ and SIVneg mangabeys. Previous transcriptome studies following SIV infection of mangabeys identified differential, but transient gene expression changes during the acute phase of the infection that later resolve in the chronic phase [9]. Therefore, our data support the idea that chronic immune activation may be hindering cellular responses to opportunistic pathogens during chronic HIV infection, but not during SIV infection of natural hosts.

When comparing BCG responses in humans compared to those of mangabeys, we observed an increase in IL-17 gene expression that was unique to mangabeys. Because IL-17 production promotes containment and protective immunity against mycobacteria [52–54], this may be a species-specific early response to BCG that helps to promote mycobacterial killing in sooty mangabeys. While our analysis did not determine the cellular source of IL-17 in the mangabeys, we hypothesize that innate-acting non-conventional T cells may be involved. Indeed, IL-17 production has previously been shown to originate from gamma-delta (γδ) T cells and double negative (CD4-CD8-) T cells, rather than CD4 T cells in naïve and Mtb-infected mice [55]. Additionally, we have previously shown that sooty mangabey double negative (DN) T cells can produce IL-17 to a similar level as bulk CD4+ T cells, and that this capacity remains unchanged during SIV infection [56].

IL-6 is one of the primary cytokines elicited from mycobacteria-infected monocytes [57]. In our 248 gene analysis, IL-6 was the most strongly upregulated gene transcript in human PBMC in response to BCG, and it was upregulated 1.5x higher in HIV+ donors compared to HIVneg. This finding is supported by published clinical data demonstrating that HIV/pulmonary Mtb co-infected individuals have higher levels of several cytokines, including IL-6, when compared to persons infected with only Mtb [58]. Previous *in vitro* studies suggest that IL-6 production from infected monocytes effectively prevents potent IFN-γ host responses, inhibiting bacterial control during mycobacteria infections [59]. We therefore speculate that the robust IL-6 response observed in humans, particularly HIV+ donors, contribute to the less robust IFN-γ upregulation following exposure to BCG. In contrast to humans, BCG-exposed mangabey PBMC upregulated IL-6 to only 10% of that of humans, regardless of SIV status. In addition, SIV+ mangabeys maintained robust IFN-γ transcript upregulation following with BCG exposure (Fig 6).

Taken together, our findings support a model in which increased baseline immune activation in HIV+ donors associates with alteration of the earliest innate responses to BCG, including differences in: 1) whole blood monocyte cytokine production and 2) transcriptional regulation of key genes that represent important components to the immune response to mycobacteria. Here we identified BCG response signatures that become altered during HIV infection, and that that are distinct from maintained immune response to BCG associated with non-pathogenic SIV infections. Therefore, these immune protein and gene signatures represent potential mechanisms by which pathogenic HIV infection alters innate immune responses, leading to susceptibility to mycobacterial coinfections. Future studies could exploit these differences to evaluate immune therapeutic approaches to improve immune responses to BCG and other opportunistic pathogens in HIV-infected individuals.

## Supporting Information

S1 Fig Flow cytometry fluorescence minus one (FMO) plots. Flow cytometry gates for the intracellular cytokine assays were defined by FMO staining. Representative flow cytometry plots and gating strategy of cytokine-producing monocytes, which were first defined as being live and CD3 negative (not shown), CD14+, and producing TNF-α (left side) or IL-12 (right side) following 6h exposure to BCG. The top plots demonstrate cytokine staining when the full panel is used, while the bottom plots demonstrate staining in the absence of either TNF-α APC (left) or IL-12 PE (right).
(TIF)

S2 Fig Percentage of whole blood monocytes producing TNF-α in response to increasing doses of BCG. Intracellular cytokine staining was used in conjunction with flow cytometric analysis to measure TNF-α production by human or mangabey monocytes. Whole blood was stimulated with increasing doses of *M*. *bovis* BCG for 6h in two uninfected humans (solid bars) or mangabeys (patterned bars) in order to determine the optimal dose for evaluating BCG-induced cytokine production (different multiplicities of infection (MOI)s for BCG are indicated, MOI of 10 was utilized for the experiments described). Whole blood cells were first gated on live, CD3neg, CD14+ cells before gating on the percentage of TNF-α+ monocytes. Error bars represent the standard deviation the mean calculated from triplicate assays.
(TIF)

S3 Fig Monocytes from HIV-infected donors upregulate IL-12 in response to LPS. The percentage of IL-12-producing monocytes (defined as CD14-bright, CD3-SSC-mid cells in PBMC) following 6h LPS stimulation was assessed via flow cytometry (as shown in Figs 1 and S1) in HIVneg (unfilled circles) and ART-naive HIV+ (filled circles) donors. Lines represent the medians for each group (*p<0.05, **p<0.01 Mann-Whitney).
(TIF)

S4 Fig Example of genes that are differentially expressed in PBMC controls (without BCG) in HIV+ and HIVneg humans (p<0.05 after correction for multiple comparisons). The NK cell-mediated cytotoxicity pathway was significantly enriched by genes more highly expressed at baseline by HIV+ donors in our dataset (A). Other genes known to be altered during HIV infection, including KI67, CCL5, and IL-12RB1 were also found to be differentially expressed between HIVneg and HIV+ donors in our dataset (B). The cytokine-cytokine receptor interaction pathway was enriched by genes expressed to a significantly lower extent by HIV+ donors in our dataset (IL-7R, B, last panel). Log copy number of RNA molecules is displayed on the y-axis; the line represents the median for each group (*p<0.05; **p<0.01; Mann-Whitney).
(TIF)

S1 Table Kyoto Encyclopedia of Genes and Genomes (KEGG) classification of genes differentially expressed in unstimulated samples between HIVneg and HIV+ Donors. Utilizing KEGG classification the gene transcripts associated with NK cell mediated cytoxicity were increased in HIV+ donors. In addition, gene transcripts associated with cytokine-cytokine receptor interaction were decreased in HIV+ donors. Genes that represent each pathway and statistics are presented.
(DOCX)
