# Supplementary figures and images for: Nonpathogenic SIV and Pathogenic HIV Infections Associate with Disparate Innate Cytokine Signatures in Response to Mycobacterium bovis BCG
Source: PLoS One. 2016 Aug 9;11(8):e0158149. doi: 10.1371/journal.pone.0158149 (PMC4978473; doi:10.1371/journal.pone.0158149)

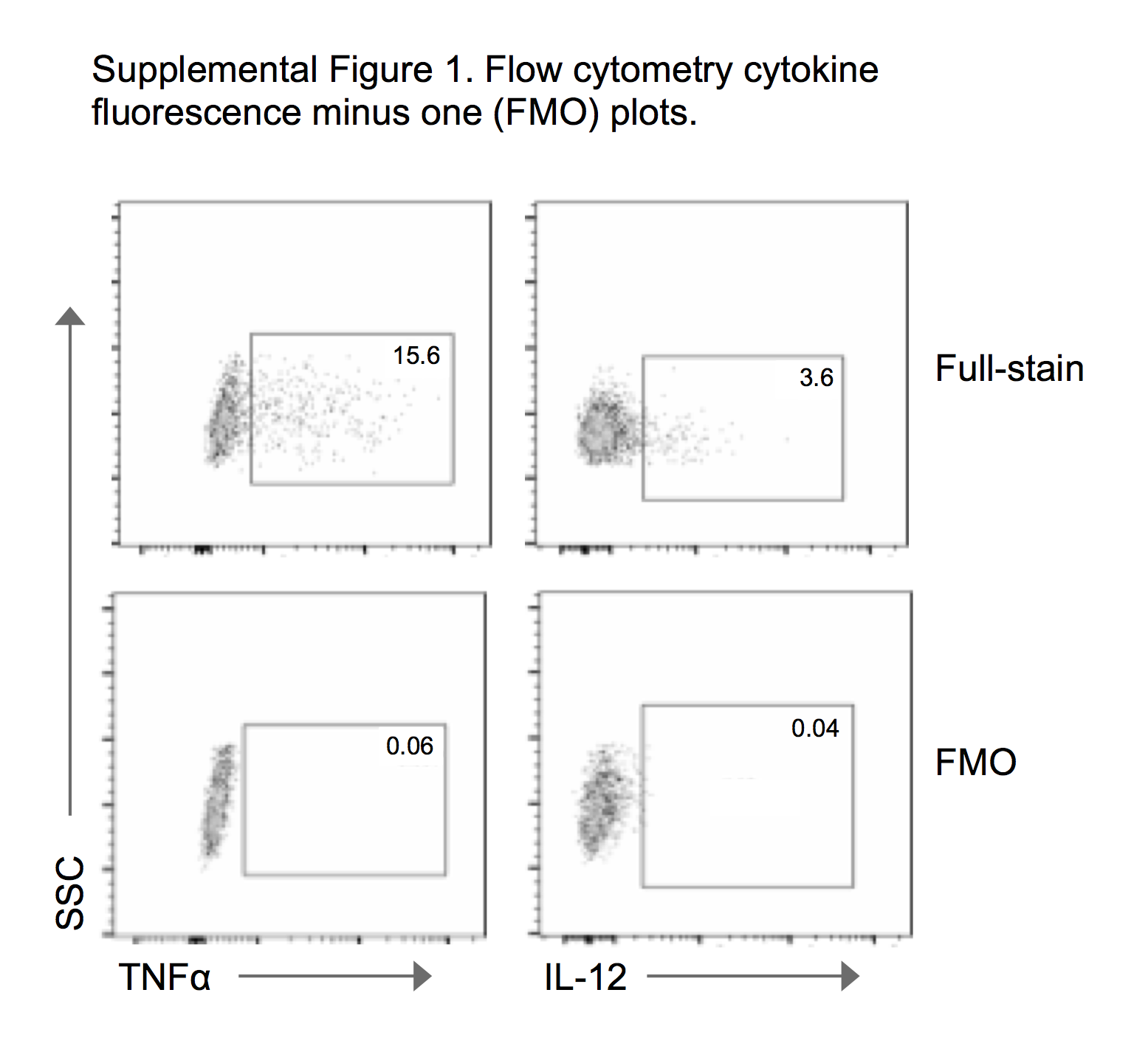

Supplement: S1 Fig — Flow cytometry gates for the intracellular cytokine assays were defined by FMO staining. Representative flow cytometry plots and gating strategy of cytokine-producing monocytes, which were first defined as being live and CD3 negative (not shown), CD14+, and producing TNF-α (left side) or IL-12 (right side) following 6h exposure to BCG. The top plots demonstrate cytokine staining when the full panel is used, while the bottom plots demonstrate staining in the absence of either TNF-α APC (left) or IL-12 PE (right). (TIF) [file pone.0158149.s001.tif]

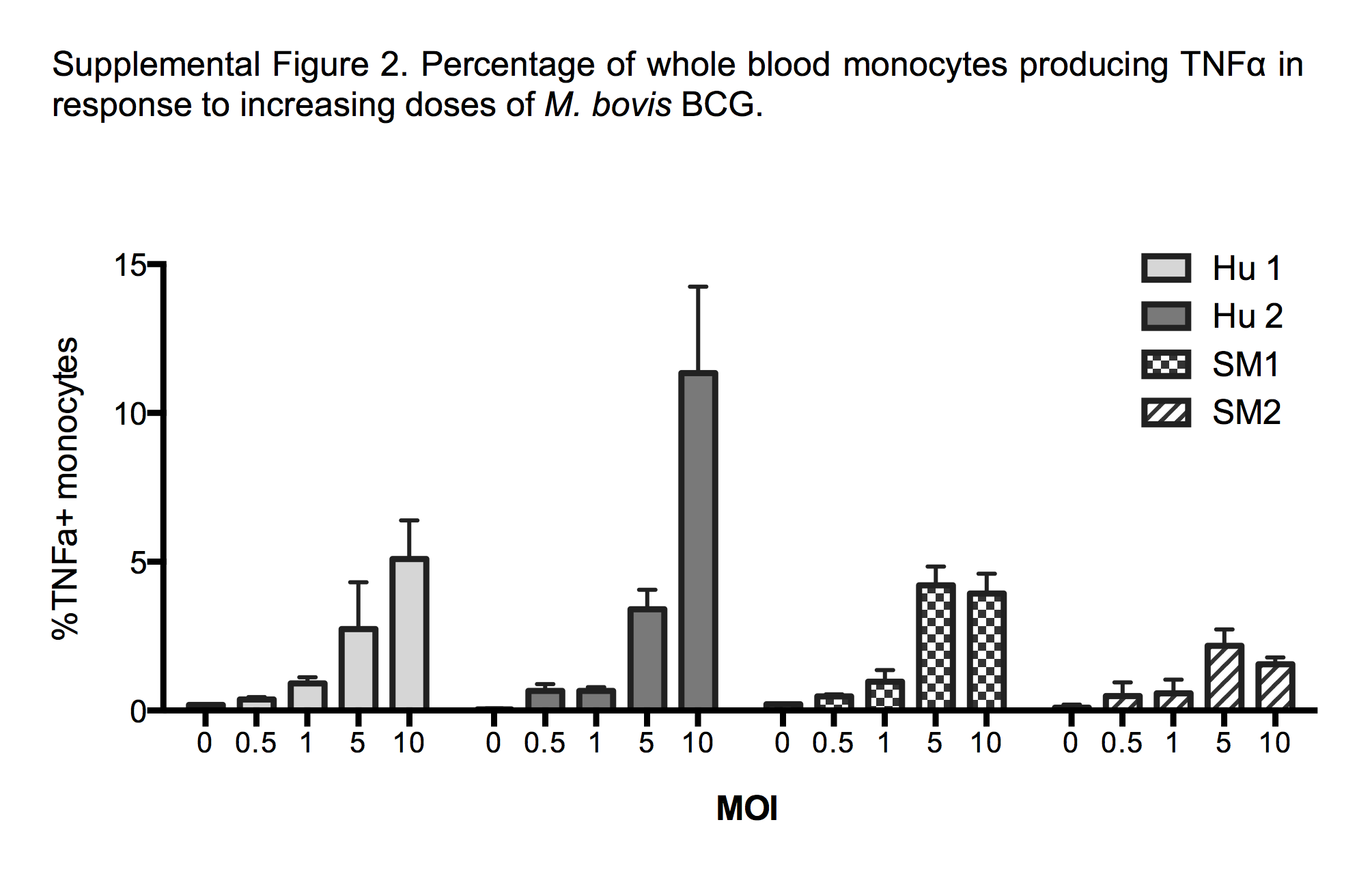

Supplement: S2 Fig — Intracellular cytokine staining was used in conjunction with flow cytometric analysis to measure TNF-α production by human or mangabey monocytes. Whole blood was stimulated with increasing doses of M. bovis BCG for 6h in two uninfected humans (solid bars) or mangabeys (patterned bars) in order to determine the optimal dose for evaluating BCG-induced cytokine production (different multiplicities of infection (MOI)s for BCG are indicated, MOI of 10 was utilized for the experiments described). Whole blood cells were first gated on live, CD3neg, CD14+ cells before gating on the percentage of TNF-α+ monocytes. Error bars represent the standard deviation the mean calculated from triplicate assays. (TIF) [file pone.0158149.s002.tif]

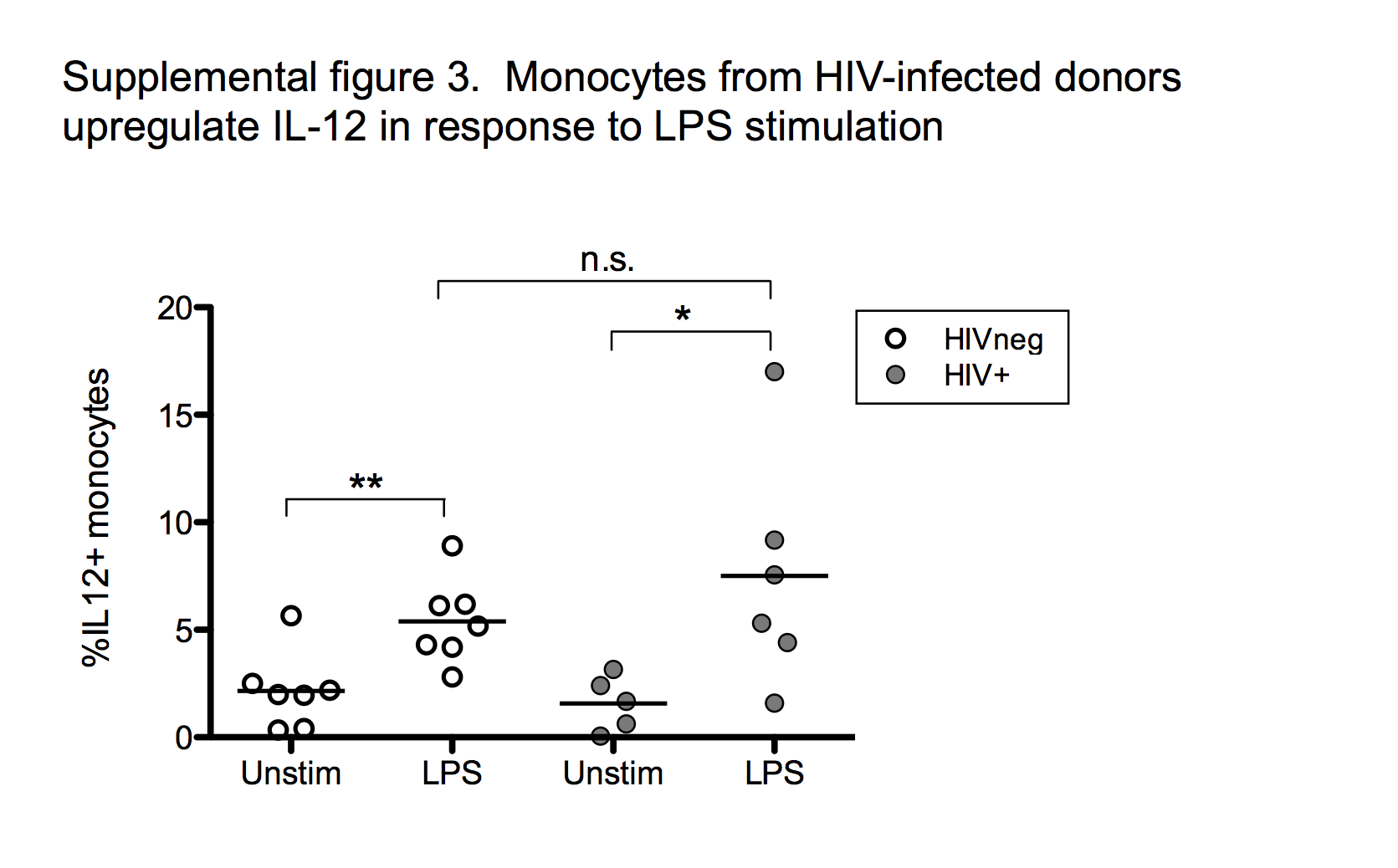

Supplement: S3 Fig — The percentage of IL-12-producing monocytes (defined as CD14-bright, CD3-SSC-mid cells in PBMC) following 6h LPS stimulation was assessed via flow cytometry (as shown in Figs 1 and S1) in HIVneg (unfilled circles) and ART-naive HIV+ (filled circles) donors. Lines represent the medians for each group (*p<0.05, **p<0.01 Mann-Whitney). (TIF) [file pone.0158149.s003.tif]

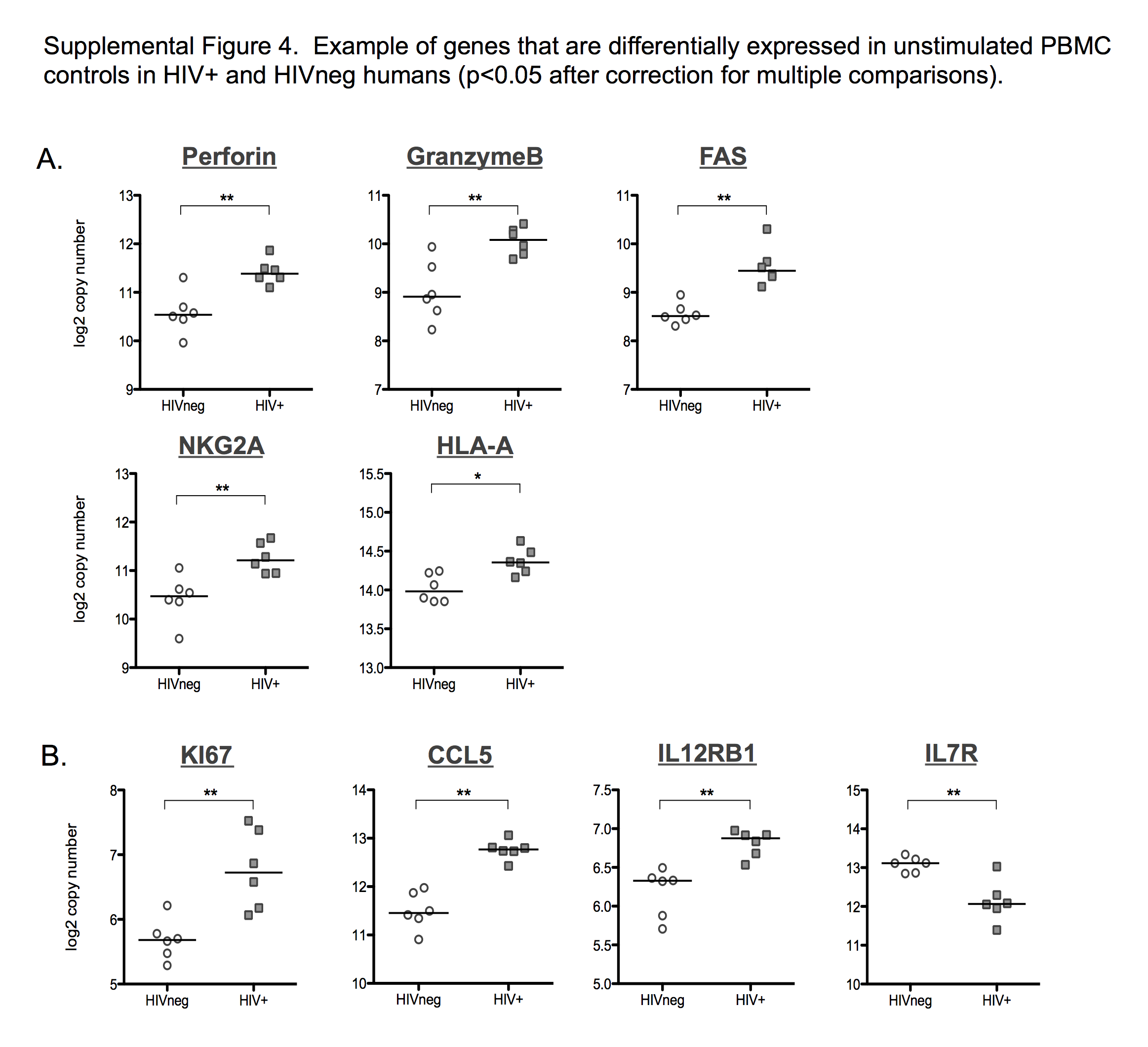

Supplement: S4 Fig — The NK cell-mediated cytotoxicity pathway was significantly enriched by genes more highly expressed at baseline by HIV+ donors in our dataset (A). Other genes known to be altered during HIV infection, including KI67, CCL5, and IL-12RB1 were also found to be differentially expressed between HIVneg and HIV+ donors in our dataset (B). The cytokine-cytokine receptor interaction pathway was enriched by genes expressed to a significantly lower extent by HIV+ donors in our dataset (IL-7R, B, last panel). Log copy number of RNA molecules is displayed on the y-axis; the line represents the median for each group (*p<0.05; **p<0.01; Mann-Whitney). (TIF) [file pone.0158149.s004.tif]
