# Supplementary material for: Nonpathogenic SIV and Pathogenic HIV Infections Associate with Disparate Innate Cytokine Signatures in Response to Mycobacterium bovis BCG
Source: PLoS One. 2016 Aug 9;11(8):e0158149. doi: 10.1371/journal.pone.0158149 (PMC4978473; doi:10.1371/journal.pone.0158149)
Supplement: S1 Table — Utilizing KEGG classification the gene transcripts associated with NK cell mediated cytoxicity were increased in HIV+ donors. In addition, gene transcripts associated with cytokine-cytokine receptor interaction were decreased in HIV+ donors. Genes that represent each pathway and statistics are presented. (DOCX) [file pone.0158149.s005.docx]

**S1 Table.**

|  | **Pathway Name** | **#Genes** | **Gene** | **Statistics** |
| --- | --- | --- | --- | --- |
| Increased in HIV+ donors (22 genes) | NK cell-mediated cytoxicity | 10 | KLRD1, ITGAL, HLA-B, CASP3, FASLG, GZMB, PRF1, HLA-A, FCGR3A, FAS | C=37;O=10;E=3.31;R=3.02; **rawP=0.0003;adjP=0.007** |
|  | Unclassified | 12 | SLPI, CCL5, MKI67, APOBEC3H, CD8A, TBX21, KLRG1,CXCR3, APOBEC3G, IL12RB1, APOBEC3F | N/A |
| Decreased in HIV+ donors (7 genes) | Cyokine-cytokine receptor interaction | 6 | CCL28, CD40LG, IL7R, CCR7,CXCR7, CCR2 | C=85;O=6;E=2.42;R=2.48; **rawP=0.0076;adjP=0.030** |
|  | Unclassified | 1 | SLC22A17 | N/A |
